# Non-uniform weighting of local motion inputs underlies dendritic computation in the fly visual system

**DOI:** 10.1038/s41598-018-23998-9

**Published:** 2018-04-10

**Authors:** Ohad Dan, Elizabeth Hopp, Alexander Borst, Idan Segev

**Affiliations:** 1 Department of Neurobiology, the Hebrew University of Jerusalem, Jerusalem, 91904 Israel; 2 Edmond and Lily Safra Center for Brain Sciences, the Hebrew University of Jerusalem, Jerusalem, 91904 Israel; 3 Department of Circuits-Computation-Models, Max-Planck-Institute of Neurobiology, Am Klopferspitz 18, 82152 Martinsried, Germany

## Abstract

The fly visual system offers a unique opportunity to explore computations performed by single neurons. Two previous studies characterized, *in vivo*, the receptive field (RF) of the vertical system (VS) cells of the blowfly (*calliphora vicina*), both intracellularly in the axon, and, independently using Ca^2+^ imaging, in hundreds of distal dendritic branchlets. We integrated this information into detailed passive cable and compartmental models of 3D reconstructed VS cells. Within a given VS cell type, the transfer resistance (TR) from different branchlets to the axon differs substantially, suggesting that they contribute unequally to the shaping of the axonal RF. Weighting the local RFs of all dendritic branchlets by their respective TR yielded a faithful reproduction of the axonal RF. The model also predicted that the various dendritic branchlets are electrically decoupled from each other, thus acting as independent local functional subunits. The study suggests that single neurons in the fly visual system filter dendritic noise and compute the weighted average of their inputs.

## Introduction

In the last few decades, detailed cable and compartmental models have been constructed for a variety of neuron types. These experimentally-based models provided key insights into the mechanisms governing the input/output repertoire of neurons and their computational capabilities (reviews in^1–4^). Neurons, the fundamental information processing units of the brain, receive their (analog) input via dendritic synapses and produce an axonal output, typically in the form of a train of spikes. However, understanding neurons as input/output computation devices, is limited in most cases since, for technical reasons, synaptic input e.g., during sensory processing, cannot be characterized whereas the axonal output is more readily available. Namely, experimentally, we typically have only “half of the picture” – the output is known but the input is unknown (see however recent advances^5,6^).

This missing information is tackled in an extensive body of theoretical studies, whereby the input/output properties of single neurons are explored. In this framework, detailed realistic models of neurons with dendrites and distributed synapses are constructed, and then their repertoire of input/output relationships is examined. Classical examples are e.g., for hippocampal Ca1 Neurons^7^; cortical pyramidal neurons^8^ and the cerebellar Purkinje cells^9^; See also the pioneering theoretical papers by W. Rall^10–12^, theoretical study by Agmon-Snir *et al*.^13^, and reviews^2,4^.

A rare exception in regard to experimentally available data are the lobula plate tangential cells of the fly visual system, in which both the input^14^ and the output^15^ have been characterized, for the same neuron type. The fly’s visual processing path starts at the retina and continues in a columnar fashion. The retina is composed of several hundreds of ommatidia^16^ each comprising eight photoreceptors, whose axons project to downstream layers: the lamina, medulla, lobula, and lobula plate^17^. In each hemisphere, the lobula plate houses about 60 tangential cells, which are all motion-sensitive^18,19^. These cells in turn receive their input from T4 and T5 cells^20,21^, collecting hundreds to thousands of inputs, which impinge on their branched dendrites, ramify throughout the neuropil^22–26^ and innervate downstream motor units^27^. Based on their overall preferred direction^27^, lobula plate tangential cells are divided into several subgroups – *inter alia*, the horizontal system and the vertical system (VS) cells^19,28^. The latter consists of a group of 10 neurons^19,22^, with prototypical morphology^29^, whose dendrites are aligned along the dorso-ventral axis of the lobula plate. These neurons are sequentially numbered 1 to 10, from the most lateral (VS1) to most proximal (VS10)^17^.

**Figure 1:**
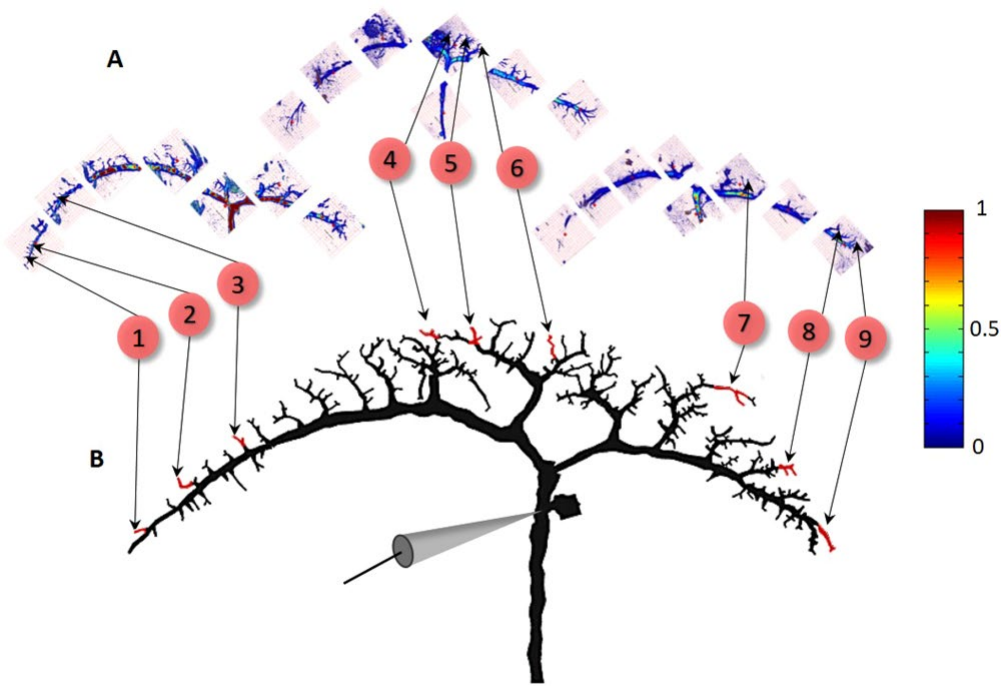
Superposition of Ca^2+^ images from dendritic branchlets of different VS cells onto a modeled prototypical cell. (**A**) Example of Ca^2+^ images from sub-trees from three different VS4 cells. These images were taken from multiple regions of interest (ROI, 155 μm × 155 μm pink squares). These ROIs show the projection of the maximal Ca^2+^ signal at rest, across 50–60 Z-plain stacks (2 μm thick each) of the lateral-proximal plane. Within each ROI, a particular imaged branchlet was selected (numbered) and mapped into a respective branchlet in a prototypical modeled cell; this process is valid due to the close similarity of the dendritic structure of VS cells belonging to the same type (see Methods). Color bar on the right denotes fluorescence measure of basal Ca^2+^ levels normalized to maximal fluorescence. White background in the figure represents Ca^2+^ below 1.5%. (**B**) Superposition of multiple recording loci from the three VS4 cells shown in A onto a prototypical reconstructed and modeled VS4 cell. Electrode in the figure shows the axonal location of intracellular voltage measurements.

**Figure 2:**
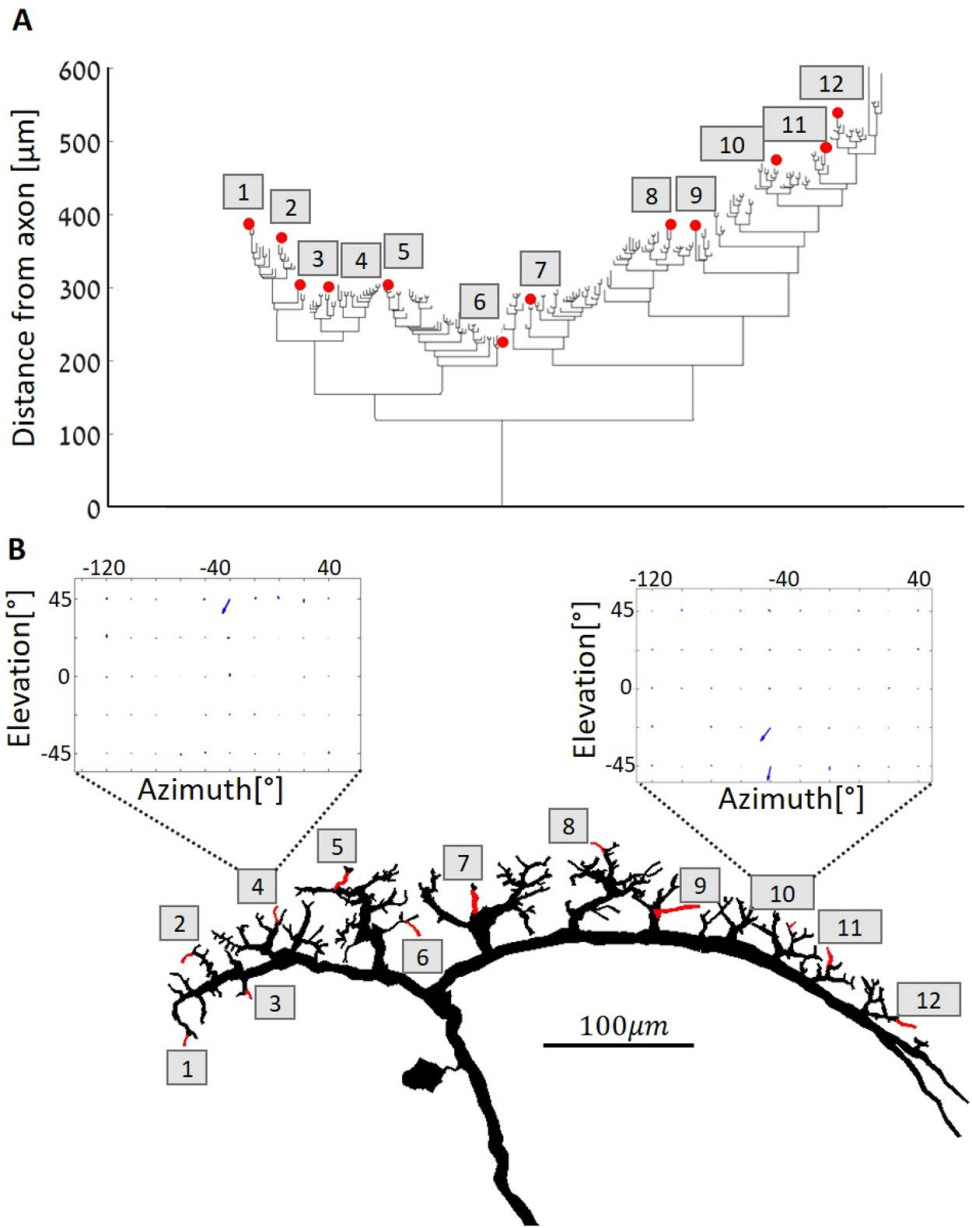
Morphological dendrogram and examples for branchlets’ receptive fields in a VS3 cell. (**A**) VS3 branching pattern. This cell consists of more than 200 terminals (dendritic tips) that span the distance of up to 600 μm from the axonal measurement point (see Fig. 1). (**B**) Reproduced from Hopp and colleagues^14^. Morphological reconstruction of VS3 is shown at the bottom, with some pivotal branchlets numbered and colored in red, corresponding to the numbered branchlets depicted at top. Insets are examples of dendritic receptive fields measured from two dendritic branchlets (#4 and #10). Arrows denote local preferred direction at the dendritic branchlet. Note that, out of the 50 vectors (blue arrows), only one large vector is apparent at the left receptive field and two large vectors – in the right receptive field (see Methods).

In recent years, the receptive fields of VS cells of the blowfly (*calliphora vicina*) were characterized in great detail by means of *in vivo* intracellular recordings from the axon during visual stimuli^15,30–32^. VS cells’ axons respond to specific patterns of visual stimuli with different preferred directions and different sensitivities in each location of the receptive field^15,33^. In a recent study^14^, the view from the axon was complemented by a view from the dendrites of VS cells (see Methods and Fig. 1A) by performing Ca^2+^ imaging of multiple dendritic branchlets from a single VS cell (in case in point, thin distal branches, typically 4th order or above, as highlighted in red in Fig. 2B), while visual stimuli were presented. Having both the dendritic and the axonal measurements at a single VS-cell provides a unique opportunity to explore the principles that govern the transformation of the cells’ multiple inputs to the axonal output, that is, the process in which the receptive fields of multiple dendritic branchlets are integrated to the axonal receptive field.

A possible scenario in this regard would be that the axonal receptive field results from a linear sum of all the dendritic receptive fields. A computation to test this option was performed by Hopp and colleagues^14^ (see also^34^). The resulting linear summation of the dendritic receptive fields was similar to that at the axon, but the match was not complete. To obtain a more accurate picture of the I/O function of VS cells, we created detailed cable and compartmental models based on the 3D reconstruction of VS cells, and incorporated into them data regarding the dendritic and axonal receptive fields obtained in experiments. We demonstrate that unequal weighting of dendritic branchlets as well as linear non-linear filtering are two functions that are likely to be implemented by the dendrites of VS cells and, interestingly, that the branchlets of VS cells operate as essentially independent local functional subunits with little interaction among them. The significance of our results are elaborated in the Discussion, which also outlines trajectories for future research to achieve a better understanding of the I/O characteristics of VS neurons.

## Results

### Superposition of *Ca*^*2+*^ images from multiple dendritic branchlets and cells onto a prototypical VS model

To generate the most detailed model of VS cells’ dendritic orientation preferences, the receptive field for as many branchlets as possible per cell type is desirable. However, the reconstruction of even a single branchlet’s receptive field is a tedious and prolonged process; this difficulty is further exacerbated by the limited lifetime of each sampled cell. Thus, the total number of dendritic branchlets receptive fields sampled from a single cell by Hopp and colleagues^14^ was limited to about 13 branchlets/cell. To overcome this limitation, we superimposed measurements, in the form of Ca^2+^ images, obtained from several cells of a given type (e.g., VS4) onto a single prototypical cell of that same type (Fig. 1). Specifically, the imaged cells (Fig. 1A) were overlaid on a reconstructed morphology (the model, Fig. 1B), and recording sites, registered by the acquisition-microscope, were matched to respective locations on the dendritic tree of the model. This operation yielded a total of 17, 116 and 39 dendritic branchlets’ receptive fields, obtained from 2, 8 and 3 specimens of VS3, VS4 and VS5, respectively. The above procedure whereby different cells are superimposed onto a single prototypical modeled cell is justified, as VS cells of a given type in different specimens are very similar, both morphologically and in their response to directional stimuli^19,29,30^.

### Morphological and electrotonic characterization of VS cells

Table 1 summarizes the morphological characteristics of six types of VS cells.

**Table 1:** Morphological characteristics of six VS cell types obtained from 3D reconstructions. Measurements were made on prototypical VS1, VS2, VS3, VS4, VS5 and VS9 cells^23,68,75–80^. The numbers in the table were extracted directly from the 3D reconstructed morphologies using the TREES toolbox^69^.

VS type	Total dendritic length [μm]	Total membrane area [μm^2^]	Average diameter [μm]	Average terminal diameter [μm]	Number of branchlets
1	6,789	60,334	2.25	0.98	649
2	5,303	59,489	3.0	1.20	689
3	4,501	51,523	2.8	1.01	417
4	4,385	46,516	3.0	1.08	281
5	4,148	35,366	1.9	0.68	519
9	2,496	32,522	3.8	1.14	141
Mean	4,604	47,625	2.89	1.02	449
SD	1,289	10,778	0.59	0.18	194

The cells are intensely branched, with an average of 449 ± 194 (SD) dendritic branchlets per cell. The dendrites are rather extended, with total dendritic length of several thousand micrometers. The cells are relatively thick (a few μm in diameter), with a total membrane area of several tens of thousands μm^2^.

One concrete example to the data shown in the table is shown in Fig. 2A. The figure shows the morphological dendrogram of a reconstructed VS3 cell, and Fig. 2B – its anatomy. In Fig. 2A, the 12 branches from which the receptive fields were measured following visual input are marked in red. The cell itself comprises more than 200 dendritic terminals that span the distance of up to 600 μm from the axonal measurement point (see Fig. 1). Note that some dendritic branchlets (e.g., #6) end much closer to the axon than others. In fact, the entire left trunk (Branchlets #1-#6) is closer to the axonal measurement point than the right one. This variance in distance might affect the extent to which the inputs of different dendritic branchlets affect the receptive field of the axon (see below). Figure 2B depicts the 3D-reconstructed VS3 cell, with the numbered branchlets corresponding to those in Fig. 2A, and with two examples of the local dendritic receptive fields in the insets. The receptive fields are depicted by 50 vectors per branchlet receptive field, most of which are very small, as illustrated by very small blue arrows. As was shown by Hopp and colleagues^14^, all branchlets in the VS system display a clear and distinct direction selectivity; e.g. for the examples shown in the figure, Branchlet #4 responds strongly to visual motion at elevation of 45° and azimuth of −30°, whereas Branchlet #10 is sensitive to the elevations of −45° and −22.5 at azimuth −50°. Indeed, dorsal branchlets (in case in point, those on the left trunk) respond to stimuli in the upper part of the visual space, whereas ventral branchlets (those on the right trunk) respond to stimuli at lower elevations. These local dendritic receptive fields are then likely summed up at the axon, shaping the output receptive field of the cell^14^.

**Figure 3:**
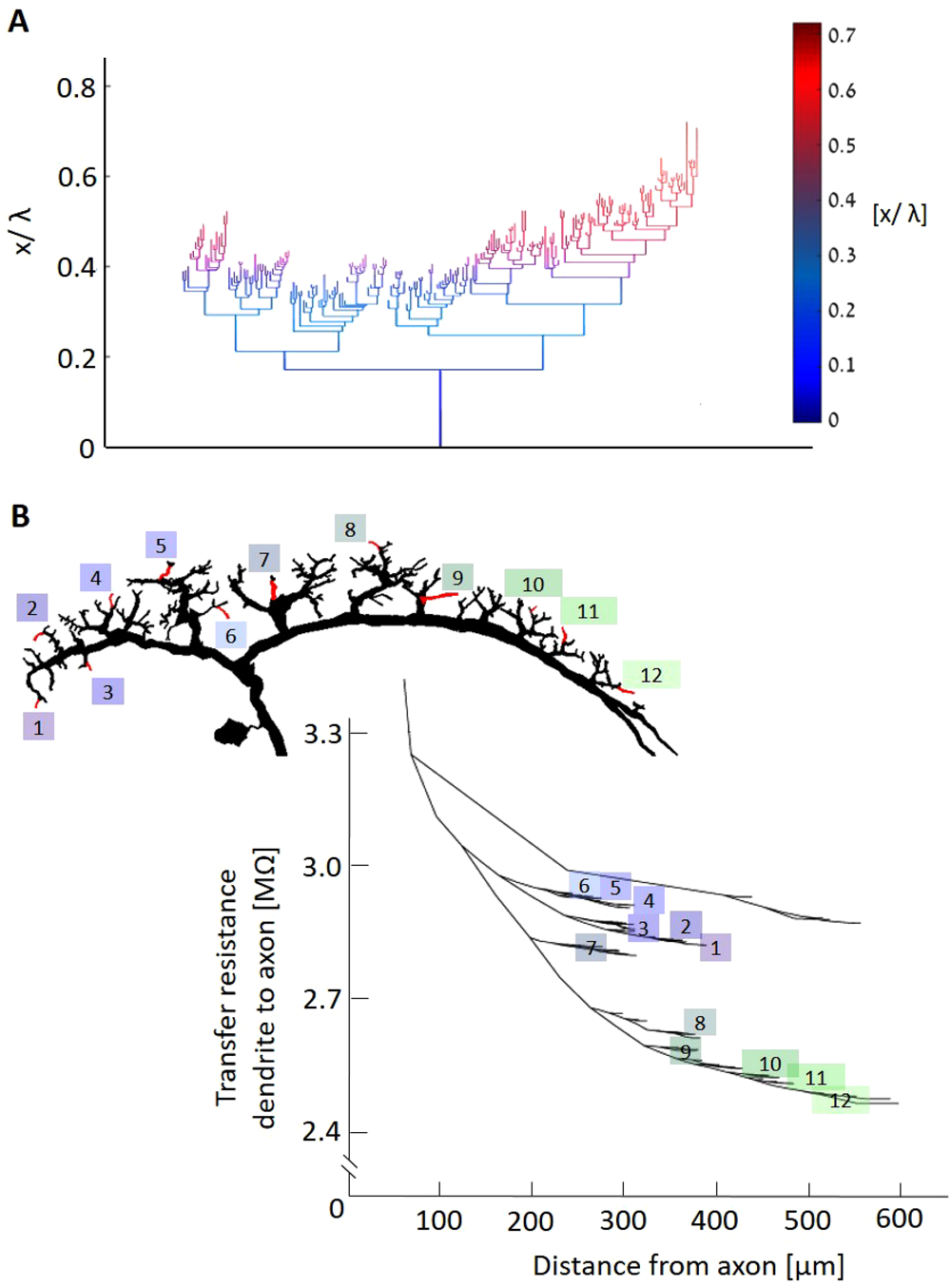
Cable analysis of a VS3 cell. (**A**) Electrotonic dendrogram, in dimensionless units of x/λ. Distal branchlets are ~0.6 λ from the axonal measurement point. (**B**) Transfer resistance (V_axon_/I_dend_) between each dendritic locus and the axon. The input resistance at the axon (V_axon_/I_axon_) was 4.6 MΩ (measured from the model using NEURON, not shown). Parameters used were R_m_ = 2,000 Ω·cm^2^, R_i_ = 40 Ω·cm^23^.

Figure 3 displays the analysis of the electrotonic properties of a VS3 cell. These cable properties largely determine the processing of electrical signals by neurons^10^. To unravel the mechanism of dendrite-to-axon signal transfer, for each dendritic branchlet, we calculated the electrotonic distance, which is the physiological length, x, normalized by the space constant λ^10,35–37^, where λ = $\sqrt{{r}_{m}/{r}_{i}}$; *r*_*m*_ and *r*_*i*_ are the membrane and the longitudinal resistance per unit length, respectively (as defined by Rall’s one-dimensional passive cable equation, see equation (1) in the Methods). λ (in units of cm) governs, to some extent, the attenuation of voltage along the cylindrical cable (see Equation (1)). The average electrotonic distance of all terminals to the axonal measurement points of VS #1, 2, 3, 4, 5 and 9 was 0.83 ± 0.15, 0.37 ± 0.16, 0.71 ± 0.15, 0.54 ± 0.13, 0.84 ± 0.14 and 0.60 ± 0.22, respectively. As a rule, shorter electrotonic distance implies a more compact neuron, in the sense that less voltage attenuation occurs in the soma-to-dendrite direction – but not necessarily in the dendritic-to-soma direction, where such attenuation is heavily impacted by the boundary conditions at the branch-points^38^. An example of an electrotonic dendrogram for a VS3 cell is presented in Fig. 3A, which displays each branchlet’s electrotonic distance from the axonal measurement point. As in the morphological dendrogram, some branchlets are closer electrically to the axon than others, suggesting that the various branchlets contribute differently to the shaping of the axonal receptive field.

A key parameter for estimating the contribution of a given branchlet to the voltage response at the axon is the transfer resistance from the branchlet to the axon, which is computed as the ratio between current injected at the branchlet (*I*_*branchlet*_) and the corresponding voltage change developed at the axon (*V*_*axon*_)^39^. This was calculated for the steady-state case, in Fig. 3B, for the VS3 cell shown in Fig. 3A. The diverse transfer resistance from the various branchlets in Fig. 3C implies that the contribution of the receptive field of individual branchlets to the overall axonal receptive field is not uniform (ranging up to approximately 20%; e.g., transfer resistance of branchlet #6 is 2.92 MΩ and of branchlet #12 is 2.47 MΩ). It stands to reason, then, that the contribution of each branchlet to the axonal output should be weighted, as a first approximation, by its transfer resistance (see below). An underlying assumption to this statement is that each branchlet receives similar synaptic input during visual processing (see Discussion).

### Dendritic branchlets are electrically decoupled from each other

To evaluate the impact of dendritic inputs on the axonal output we computed the transfer resistance from each branchlet to the axon (Fig. 3B). However, as the neuron constitute a single connected dendritic tree, rather than a conglomeration of multiple isolated input channels, we next examined the electrical “cross talk” among dendritic branchlets. In other words, we checked the impact of the receptive field of one branchlet on that of others.

**Figure 4:**
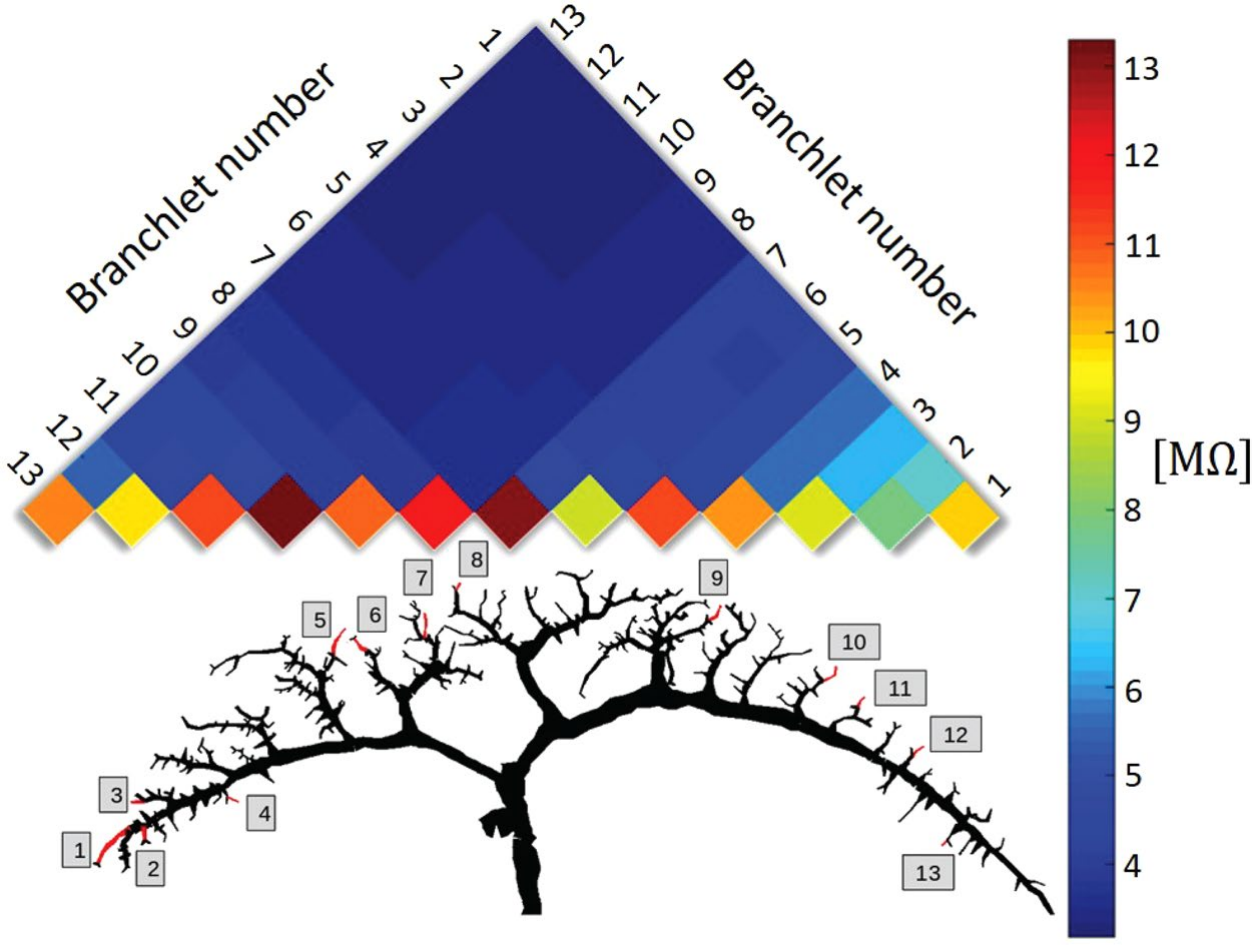
Dendritic branchlets are electrically decoupled from each other. Transfer resistance (in MΩ), coded in colors, between 13 branchlets of the modeled VS4 cell is shown at bottom (the 13 branchlets in the reconstructed tree are depicted in red). The colored squares at the lower part of the triangular matrix represent the input resistance at the respective branchlets (measured from the model using NEURON). The transfer resistance between any two branchlets falls into the zones coded as blue for the most part, suggesting that the transfer resistance value is relatively small and that the various branchlets are electrically decoupled from each other. The electrical parameters are as in Fig. 3. Note that, in a passive system, the transfer resistance measure is symmetrical: transfer resistance (i, j) = transfer resistance (j, i); thus, the total of the 13 × 12 combinations of the transfer resistance values could be compactly represented by the triangular (13 × 6 unidirectional) values.

To assess these putative cross-branchlet influences, we selected a set of 13 branchlets of a modeled VS4 cell (Fig. 4, bottom) that were morphologically representative in the sense that they were uniformly spread over the retinotopic span of the dendritic tree^14^. We then calculated the transfer resistance between all pairs of these branchlets (Fig. 4, top). The typical input resistance of the branchlets (the lower colored row in Fig. 4) ranged between 8 and 13 MΩ. In contrast, the transfer resistance between the branchlets was much lower, ranging from 3 to 4 MΩ (blue region in Fig. 4). This indicates that the various branchlets composing a VS4 dendritic tree are, to a large degree, electrically decoupled from each other, and that accordingly, to a good approximation, the effect of branchlets on each other is small as compared to the local input resistance at a given branchlet. It can therefore be plausibly concluded that the receptive field at each branchlet is determined mostly by its local cable properties and is independent of the receptive fields of other branchlets. It must also be pointed out that, if voltage-dependent channels were included in the model, the electrical decoupling among different branchlets would be even larger than shown in Fig. 4 (see Suppl. Figure 1 and Borst *et al*., 1996, Fig. 11). Note that, when reducing R_m_, the effect on the branch transfer resistance is greater than the effect on the input resistance (i.e. they are not reduced proportionally). This is directly expected from Rall’s cable theory. Indeed, when comparing the color bars in Fig. 4 and Fig. S1B, the largest values, representing the branchlet input resistance, is roughly 13.2 MΩ in Fig. 4 (before reducing R_m_) and only 8.7 MΩ in Fig. S1B, after reducing R_m_ (namely, the branch-input resistance was reduced by a factor of 8.7/13.2 = 0.70). The lowest values in the color bars (representing the transfer resistance between the most decoupled branchlets) are roughly 1.6 and 3.5, respectively (the transfer resistance was reduced by a factor of 1.6/3.5 = 0.48). Hence, when comparing the two dendritic trees (before and after reducing R_m_), the branchlets of the latter dendritic tree are more electrically decoupled (function as more independent subunits).

### Impact of dendritic cable properties on the axonal receptive field

**Figure 5:**
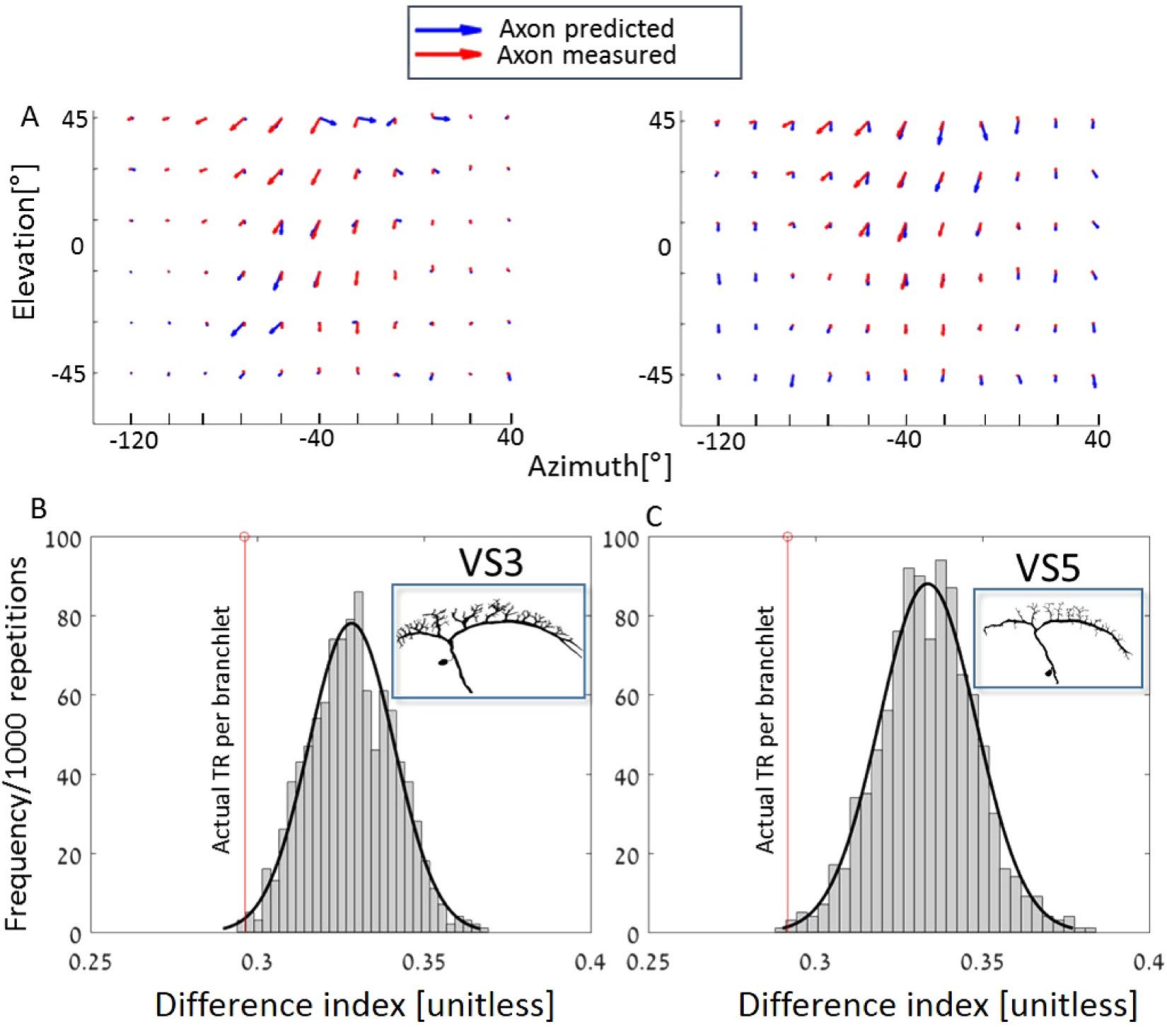
Weighting the dendritic receptive fields of each branchlet by its transfer resistance improves the match between the experimental and the predicted axonal receptive field. (**A**) The receptive field at the axon of VS5 measured experimentally (red arrows) compared to that predicted (blue arrows). The left-hand panel shows equal weighting, resulting in the difference index of 0.411. On the right, the receptive fields of all dendritic branchlets are weighted by their respective transfer resistance values, resulting in improved (albeit not perfect: see Discussion) match, with a difference index of 0.293. The modeled VS5 cell is shown at the inset of C. (**B** and **C**) The difference index for VS3 and VS5 cells (insets) computed using the actual transfer resistance values for each branchlet (vertical red lines at left), derived from the cable models of the respective cell, and using the transfer resistance values that were randomly reshuffled among branchlets (bars). Also shown is the corresponding fit to a normal distribution (see Methods).

Our results, displayed in Figs 3 and 4, suggest that the axonal receptive field is shaped by a weighted sum of the receptive fields at the different dendritic branchlets. The quality of the match (Fig. 5) between the experimental (red arrows) and the predicted (blue arrows) axonal receptive field was assessed using the difference index (see Methods). It is evident, based on Fig. 5, that the match is indeed improved when the dendritic receptive field of each branchlet is weighted by its transfer resistance as shown in Fig. 5A left, as opposed to the case of equal weighting for all branchlets, as per Fig. 5A right, with the difference index values at 0.293 and 0.411, respectively. To assess the extent of the improvement resulting from using the weighted sum, we reshuffled the weights amongst branchlets and recalculated the difference index again for each case (Fig. 5B,C, colored bars). As depicted by the red vertical lines on the left-hand of each panel, in the case where each branchlet was weighted by its corresponding transfer resistance, the difference index was clearly smaller than when the transfer resistance values were randomly distributed among the various branchlets. The discrepancy between the mean distribution in the two scenarios stands at more than 2 SDs, suggesting that the axonal receptive field is strongly affected by a transfer-resistance weighted sum of the branchlets’ receptive fields.

### Filtering dendritic noise

**Figure 6:**
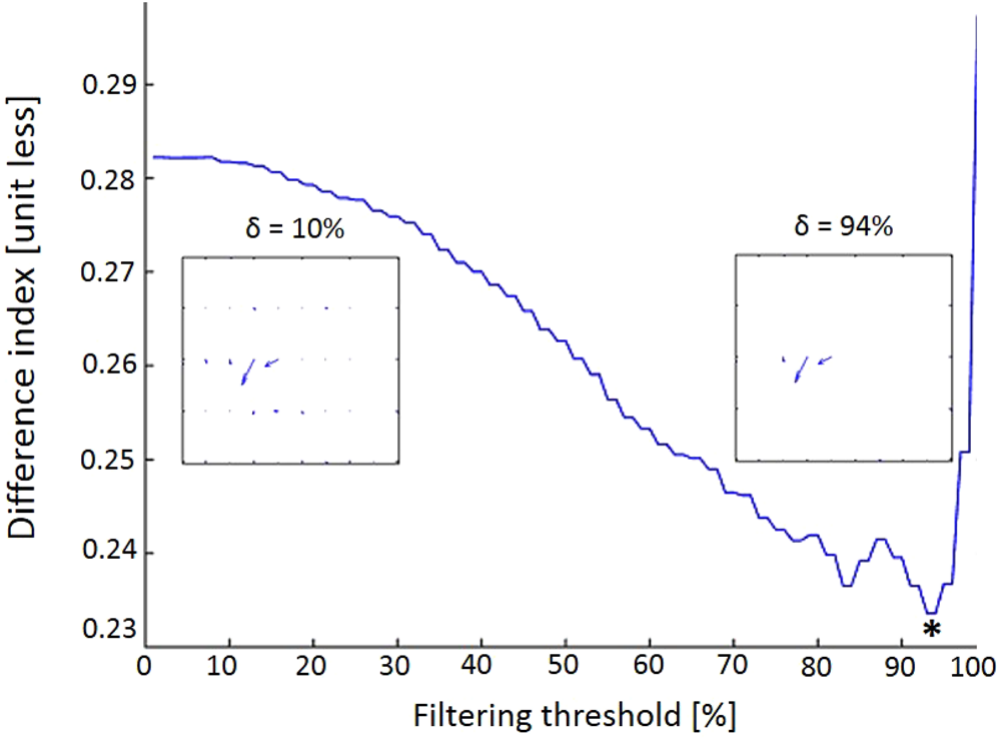
Filtering small local vectors at the branchlets’ receptive fields improved the match between the experimental and the computed axonal receptive field. The figure shows a monotonic decrease in the computed difference index (i.e., an improvement in the experimental-to-theoretical match of the axonal receptive field) when small local dendritic voltage responses were progressively eliminated, starting from no filtering (0% threshold such that all 50 vectors per branchlet’s receptive field are taken into account) and filtering up to 94% of the smallest vectors (marked by an asterisk). The insets illustrate the filtering process: at left only 10% of the smallest vectors were eliminated, as compared to 94% at right, such that only the three largest vectors were taken into account in computing the difference index.

In search of global computational principles implemented by VS dendrites, we relied on the study of Hopp and colleagues^14^. We calculated the response magnitudes at all 50 locations (designated by 50 vectors in Fig. 6 inset) within each branchlet’s receptive field and normalized each receptive field by its largest vector (which was set as 1). The latter operation enabled us to compare the distribution of vectors’ lengths across all the receptive fields.

We found that the response magnitudes of receptive fields could be divided into two distinct, uneven populations – a large number of small vectors and a few large vectors (see e.g. Fig. 2B, inset). We counted the number of small and large vectors across all the branchlets of several VS cells (VS2 through VS7): For all the cells examined, more than 95% of the total of 239 dendritic receptive fields presented with either only one or two vectors that were larger than 0.7. Furthermore, for all (but one) of the receptive fields, the response sensitivity in at least 80% of the vectors was less than 0.4. These findings point to a characterizing feature common to all dendritic receptive fields: The maximal activation response is confined to areas corresponding to one to two locations, while activation in all other locations is much less.

This observation led us to probe the role of VS cells’ dendrites in forwarding signals from low-sensitivity locations. We hypothesized that each branchlet might be “responsible” for transmitting visual information from only a restricted region of its receptive field. Specifically, we assumed that a branchlet reliably transmits signals only from a maximally-sensitive locations, while acting as a filter for signals from areas of low sensitivity (Fig. 6). Such non-linear filtering operations could be biologically implemented by active dendritic membrane processes that were shown to exist in VS cells^40^ and by nonlinear voltage-calcium relationship^41^. In addition, two recent elegant studies have shown that nonlinear phenomena are expected in thin dendrites at the sub-micron regime (<0.5 μm)^42,43^. The average diameter of the branchlets in our dataset is 1.02μm (Table 1). Still, for some thin dendrites in the VS cells, this nonlinear phenomenon should be considered in future refinements of the present model.

To test this hypothesis, we filtered out signals below a certain threshold (see Methods), thus effectively discarding signals from non-sensitive locations. We found that this further improved the match between the axonal receptive field measured and the integrated signals from all the locations of the respective dendrite, such that the difference index value decreased, up to a certain filtering threshold (marked with asterisk in Fig. 6). The optimal difference indices (DI) obtained for VS 3, 4 and 5 were 0.283, 0.236 and 0.280, respectively, which are smaller than those presented in Fig. 5, where only weighted average was computed. We note that this effect is the result of applying both filtering of small dendritic signals and weighting the input according to the transfer resistance from the dendritic branchlets to the axon. When combined, these two functions improve the axon-dendritic match. Indeed, e.g., for VS4, when applying only weighted averaging DI is reduced from 0.411 to 0.316 and when applying only filtering it is reduced from 0.411 to 0.27; when applied together the DI was reduced to 0.236.

## Discussion

More than 50 years ago W. Rall introduced the cable theory for dendrites^10,11,44^ (see recent review^4^ and other relevant studies^1,7,45–47^). This seminal work laid the foundation for theoretical and computational studies on how the spatially and temporally distributed synaptic input that impinges on the dendritic tree shapes the axonal output. However, despite impressive recent technical advances in recording dendritic activity, e.g., during sensory processing^3,5,27,48–56^, studies have provided only a partial view of the input that dendrites receive *in vivo*. A comprehensive experimental understanding of neurons as I/O devices is still lacking.

A major step in in this direction was made recently with respect to VS cells in the blowfly visual system^14^. Two-photon calcium imaging performed *in vivo* made it possible to characterize the preferred location and direction of local motion cues (the local receptive field) in dozens of dendritic branchlets of various VS cells. In addition to dendritic recordings, previous studies^15^ have also supplied *in vivo* intracellular recordings from the axon of VS cells during visual processing. The present study has created a unified dataset by taking advantage of cross animal stereotypy to combine prior data across animals into one prototypical model. Specifically, dendritic measurements from multiple experiments involving VS cell of a large number of blowflies were superimposed onto a prototypical model of a single VS cell (e.g., VS4 in Fig. 1). This process was implemented for several types of VS cells yielding up to 116 independent input dendritic loci per cell type. The data obtained afforded a comprehensive and realistic representation of the VS cells’ input map during visual processing (Fig. 1).

The extensive characterization available for both the dendritic input and the axonal output, acquired in previous studies, combined with the detailed compartmental and cable models of the 3D reconstructed VS cells developed in the present study, provided unprecedented opportunity to systematically explore the principles of dendritic computation during visual processing.

First, relying on cable theory, we characterized the VS cells’ morpho-electrotonic properties (Figs 2 and 3). We found that, for a given VS cell type, the cable distance from the distal branchlet to the axon for various branchlets ranges from 0.4 λ to 0.6 λ, the variance standing at about 30%. At the same time, the transfer resistance from the various dendritic branchlets to the axon ranges from 2.4 MΩ to 3 MΩ, that is, with the variance of up to 20%. These figures led us to conclude that different dendritic branchlets contribute unequally to the shaping of the axonal receptive fields. Likewise, based on cable theory, we calculated the transfer resistance between the various pairs of dendritic branchlets (Fig. 4, top). The input resistance of the branchlets (the lower colored row in Fig. 4) ranged between 8 and 13 MΩ, whereas the transfer resistance between branchlets emerged as much lower, ranging from 3 to 4 MΩ (blue region in Fig. 4). We inferred therefrom that the various branchlets of VS cells are, to a large degree, electrically decoupled from each other, and that consequently, each branchlet could be considered as a local visual processing module that is essentially independent of other branchlets in the same cell.

We then proceeded to demonstrate that the axonal receptive field can be fairly predicted based on the receptive fields of the branchlets, when the contribution of each individual dendritic branchlet is first weighted by its branchlet-to-axon transfer resistance, and then the weighted values for all the branchlets are summed-up (Fig. 5). Additionally, our study suggests (Fig. 6) that VS cells’ dendrites act as noise filters, by damping weak dendritic signals and transmitting to the axon only the few strongest ones^37,47,57,58^. Thus, by weighting dendritic inputs by their respective transfer resistance and filtering out dendritic signals below a certain threshold, the axonal receptive field measured experimentally could be well (albeit not perfectly) construed based on the dendritic receptive fields. We stress that the present study computed the integration of dendritic signals assuming a steady-state cable model for VS cells, i.e., without taking into account time-dependent effects. This simplification is justified, considering that effective membrane (system) time constant of the VS cells is very short (less than 2 msec)^59,60^ compared to the time-course of the input signal to VS dendrites arriving from the local presynaptic local motion detectors^59,61,62^. We note that we do not yet have the actual voltage transients (synaptic potentials) in the dendrites as the experimental measurements from the dendrites relied on the slow dendritic Ca^2+^ signals.

Although our detailed cable model for VS cells provided a reasonably good fit between the axonal receptive field measured experimentally and the corresponding theoretical receptive field computed based on the dendritic receptive fields, the overlap was not full. The discrepancy may be the outcome of inter-specimen variability. We integrated dendritic measurements from multiple specimens on the working assumption that the VS cell morphology is prototypical^29^. Yet the morphology of different animals is inevitably not identical. Another reason for the mismatch obtained is the difference between the methods by which the dendritic and the axonal receptive fields were acquired, namely, intracellular voltage recording for the axon and Ca^2+^ imaging for the dendrite (see Methods). Several studies^17,34,63^ have shown that changes in calcium concentration do indeed reflect direct excitatory inputs to VS cells’ dendrite, and that the cells linearly couple changes in calcium concentration to the membrane voltage across a physiologically relevant range^64^. Nevertheless, we stress that membrane voltage, which is the model’s fundamental parameter, is not acquired from the dendrite directly but only inferred from the dendritic calcium signals. Yet another factor which clearly contributes to the mismatch between the model and the experimental results is a network effect. Each neuron, VS2 through VS9, is connected via axo-axonal gap junctions to its two adjacent VS cells^17,34,65,66^. Estimates for the coupling coefficients between neighboring cells provided by dual recording experiments were as high as 50%^67^. The strong coupling between neighboring VS cells was shown to facilitate robust coding of flow-field parameters^60^ and enable near optimal estimates of the rotation axis from a subpopulation readout of the VS network^62^. It was also suggested^67^ that, insofar as these electrical connections are responsible for the elongated horizontal extent of the VS cells’ receptive fields, they are fundamental in shaping their axonal receptive fields as well. Hence, the input to any VS cell originates from two distinct channels: dendritic input from prior visual layers and lateral input, already processed, from the rest of the VS network. Taking into account this complex nature of input to VS cells is likely to further improve the match between the model and experimental findings, and we plan to study this network effect in the near future.

A fourth factor that accounts for the imperfect fit between the theoretical predictions and empirical results is the uniform normalization imposed on the dendritic receptive fields. In our study, the data on the dendritic receptive fields of each VS cell type examined was acquired by Hopp and colleagues from multiple branchlets of a large number and variety of cells. For each cell type, the multiple receptive fields thus mapped were superimposed on a single cable model (Fig. 1). The magnitude of the Ca^2+^ signals (reflected in the length of the vectors: see, e.g., Fig. 5A) in each branchlet may represent different values of membrane voltage. Changes in fluorescence were measured as a proportion of the basal calcium level ($\frac{{\rm{\Delta }}{\rm{f}}}{{\rm{f}}}$) – a strategy that did not allow an accurate comparison of changes of fluorescence across different branches. Following Hopp and colleagues^14^, we uniformly normalized all receptive fields such that, in each branchlet, the largest response (represented by the longest vector) was of size 1. Accordingly, when integrating the receptive fields from multiple branchlets, we had to assume that their maximal responses are essentially equal, in spite of the potentially diverse activation levels.

In summary, the study has explored the various mechanisms by which hundreds of dendritic inputs integrated by the dendritic tree shape the axonal output during visual processing. A comprehensive and fairly precise mapping of the cells input-output transformation was achieved by virtue of a unique experimental dataset, comprised of multisite *in vivo* dendritic and axonal recordings, in combination with detailed modeling of the various types of VS cells. The study sheds light on the morpho-electronic principles underlying the structure of VS cells’ dendrites and on the complex computations performed by VS cells during the processing of visual input.

## Methods

### Cable and compartmental modeling of VS cells

To assess the general morphological features of VS cells, we analyzed all publicly available 3D reconstructions of these cells, which include the detailed compartmental models of VS1, VS2, VS3, VS4, VS5 and VS9 (see Table 1^23,68^). The morphological analysis of the reconstructed models of VS cells was performed using TREES toolbox^69,70^.

The dendritic influences on the axon were simulated numerically by means of the above-mentioned compartmental models and implemented in NEURON^71,72^. Specifically, we made use of the impedance class to assess remote dendritic influences at the axon and measure inter-loci transfer resistance. The model was based on Rall’s^73,74^ one-dimensional passive cable equation:

1 $$(\frac{{r}_{m}}{{r}_{i}})\frac{{\partial }^{2}V(x,t)}{\partial {x}^{2}}-{r}_{m}{c}_{m}\frac{\partial V(x,t)}{\partial t}-V(x,t)=0$$

where V is the transmembrane potential; and r_i_ (in Ω/cm), r_m_ (in Ωcm) and c_m_ (in F/cm) are all per unit lengths. As justified in the Discussion above, in this work we used the steady-state cable model for VS cells; namely, the middle term at the left side of Eq. (1) was ignored. In the compartmental approach, the continuous cable equation is discretized into a finite set of compartments, each representing a small section of the dendritic tree. The membrane properties of such a dendritic section are grouped into a single RC element and the resistivity of the dendritic cytoplasm is lumped into a single (typically passive) axial resistivity (see details on cable and compartmental modeling for neurons^10,37,73^).

The present study represents the synaptic input impinging on the distal dendritic branchlets by a steady state current and assumes passive cable properties. The passive properties of VS cells were: specific membrane resistivity (*R*_*m*_) of 2,000 Ω·cm^2^ and axial resistivity (*R*_*i*_) of 40 Ω·cm based on measurements performed by Borst and Haag^23^. These values are used throughout the study. All cells used in the simulations may be found in the online database ModelDB via: https://senselab.med.yale.edu/modeldb/enterCode.cshtml?model=231815.

### Physiology and imaging of the dendritic tree

Ca^2+^ imaging (RF measurements) was performed in multiple branchlets of different VS cells (Fig. 1A, see also^14^). The images obtained were superimposed on a prototypical compartmental model of a morphologically reconstructed neuron (Fig. 1B). In matching the dendritic locus from the experimental samples to the model, we took into account the close similarity of the dendritic structure of VS cells of the same type. We identified visually the most similar branchlet in the imaged cells with a corresponding branchlet in the model cell and in our view, the resulting spatial jitter is rather small. To spatially cover the entire dendritic structure, 6–8 maximal projection planes were required (Fig. 1A). Each of the snapshots’ acquisition was accompanied by a registration of the acquisition’s location; to align the maximal projection planes and generate a full dendritic projection, we performed a spatial comparison of all the coordinates over the dendrite (Fig. 1A). We also measured the receptive fields at various branchlets across the dendritic tree; to identify the location of the receptive field measurement, we registered the coordinates of each location (or Region of Interest – ROI) where measurements were taken (Fig. 1). These operations: Cross-plane projections, ROI alignment and visualization; were all performed using a custom-made Matlab (R2014b) code.

### Characterizing axonal and dendritic receptive fields

We integrated the axonal receptive field as measured by Wertz and colleagues^15^ and the dendritic receptive field as measured by Hopp and colleagues^14^ into a single model. Briefly, to characterize VS cells’ axonal receptive fields of the blowfly (*calliphora vicina*), a large set of moving stimuli were presented to an awake fixed fly, and simultaneously the VS cells’ activity was electrophysiologically recorded at the axon. The object of this procedure was to identify the local preferred direction (LPD) and the local motion sensitivity (LMS) at different locations of the receptive field (See Fig. 2 inset). To this end, receptive fields were divided into multiple discrete areas, and several trials were applied to each; in each trial the response to visual directional stimulus in a specific location (e.g., top left corner of the receptive field) was measured by moving a stimulus in various directions (e.g., up, down, left, right). The LPD was determined by summing up the vectors of the activation responses in each direction. The LMS was estimated as the magnitude of the preference for one direction over the others. This process was repeated for every location of the receptive field, yielding a characterization in the form of a vector map (See Fig. 2 inset). The direction and size of the vector at each location of the receptive field correspond to the LPD and LMS, respectively. As the experiments used different recording sites along the axon, the model had to be standardized. Accordingly, in the reconstructed morphological model, the axonal measurement point was set at a specific location (see Fig. 1).

To characterize the receptive field of individual dendritic branchlets, Hopp and colleagues^14^ used Ca^2+^ imaging. These researchers discretized the fly’s visual field into 50 non-overlapping spatial locations, which cover the whole visual field. At each of these, they applied multiple directional stimuli to determine the preferred direction (the LPD, measured by the magnitude of the corresponding Ca^2+^ response) and its sensitivity relative to other locations of the same receptive field (LMS).

### Difference index

Following the above-mentioned studies by Hopp^14^ and Wertz^15^, we adopted the difference index measure to serve as an error function for comparing any two receptive fields. The difference index is the average Euclidean distances between the preferred directions in corresponding locations of two receptive fields; i.e., it is a distance function that quantifies the resemblance of two receptive fields of the same size. If two receptive fields are identical, their difference index is 0; the maximum possible difference index is by definition 2 (see Equation 2).

The difference index is calculated for two receptive fields, U and W, each discretized into *n* locations (e.g., in Fig. 5A: n = 50 arrows) corresponding to *n* vectors, each of which represents the preferred direction at that specific location. Each vector is two-dimensional, with a horizontal and a vertical component (x and y, respectively). The difference index between U and W is calculated as follows:

2 $$D{I}_{(U,W)}=\frac{{\sum }_{i=1}^{n}\sqrt{{({U}_{{i}_{x}}-{W}_{{i}_{x}})}^{2}+{({U}_{{i}_{y}}-{W}_{{i}_{y}})}^{2}}}{n}$$

### Weighted contribution of branchlets’ receptive fields

In a uniform integration model^14^, each of the dendritic inputs is weighted equally, such that their average is estimated as follows:

3 $$\frac{{\sum }_{i=1}^{n}1\,\ast \,R{F}_{i}}{n}$$

where $R{F}_{i}\,\,$is any one of the dendritic receptive fields, represented by a vector map.

Yet, in assuming that all dendritic branchlets contribute in equal measure to the axonal receptive field, the uniform integration model fails to take into account that branches differ in their electrotonic distances from the axon, and hence also in their transfer resistance to the axon. To take into account the above spatial features of VS cells, in integrating the input signals of the various branches, we first weighted them by their respective transfer resistance vis-à-vis the axon. The integration of the dendritic receptive fields of the various branches can be formalized as a linear weighted average, as follows:

4 $$\frac{{\sum }_{i=1}^{n}{w}_{i}\,\ast \,R{F}_{i}}{{\sum }_{i=1}^{n}{w}_{i}}$$

where ${w}_{i}$ is a weight assigned to the receptive field of the *i*th dendritic branchlet. In the present study ${w}_{i}$ is operationalized as the transfer resistance (*V*_*axon*_/I_*branchlet*_) computed as the voltage response in the modeled axon following the injection of steady current to the modeled dendritic branchlet. It stands to reason that branchlets which are electrotonically more distant from the axon will contribute less to the shaping of the axonal receptive field.

All statistical analyses, vector map visualizations, comparisons, and per-branchlet weighting were performed using a custom-made Matlab (R2014b) code.

### Filtering out small dendritic signals

On a physiologically plausible assumption that a VS cell’s dendrite possess non-linear mechanisms^40^ by which strong signals may be preserved (linear) while those below a certain threshold may be set to zero (nonlinear), we established a linear-nonlinear threshold function for each dendritic branchlet. Thus, for all branchlets, we defined a shared filtering threshold δ which sets a threshold Θ independently for every branchlet: The value of Θ was fixed as the δ percentile of the weakest responses.

The linear-nonlinear filter was constructed such that all signals larger than the threshold Θ were preserved whereas signals with magnitudes equal to, or smaller than, the threshold were ignored. For example, setting δ to 98 would entail that the dendrite should filter out 98% of the smallest response locations. In other words, if the response magnitudes of the locations in a receptive field range consecutively from 1 to 50 (1, 2, 3, …, 49, 50), Θ will equal 49 and all but the largest signal, with the strength of 50, would be filtered out (see Fig. 6 inset).

To test different dendritic filtration levels, we constructed several linear-nonlinear models by varying δ. We note that the case of δ = 0 is tantamount to uniform linear integration.

### Data availability statement

The neuronal models used for this research are available via the online database ModelDB (https://senselab.med.yale.edu/modeldb/) via accession number: 231815.

## Electronic supplementary material

Supplementary figures
