# Supplementary material for: Non-uniform weighting of local motion inputs underlies dendritic computation in the fly visual system
Source: Sci Rep. 2018 Apr 10;8:5787. doi: 10.1038/s41598-018-23998-9 (PMC5893613; doi:10.1038/s41598-018-23998-9)
Supplement: Supplementary file 1 — Supplementary figures [file 41598_2018_23998_MOESM1_ESM.pdf]

Non-uniform weighting of local motion inputs underlie dendritic computation in the fly visual system

Ohad Dan[1], Elizabeth Hopp[3], Alexander Borst[3] and Idan Segev[*1,2]

[1]Department of Neurobiology and [2]Edmond and Lily Safra Center for Brain Sciences, the Hebrew University of Jerusalem, Jerusalem 91904, Israel. [3]Department of Circuits-Computation-Models, Max-Planck-Institute of Neurobiology, Am Klopferspitz 18, 82152 Martinsried, Germany

*Correspondence to: *idan@lobster.ls.huji.ac.il*

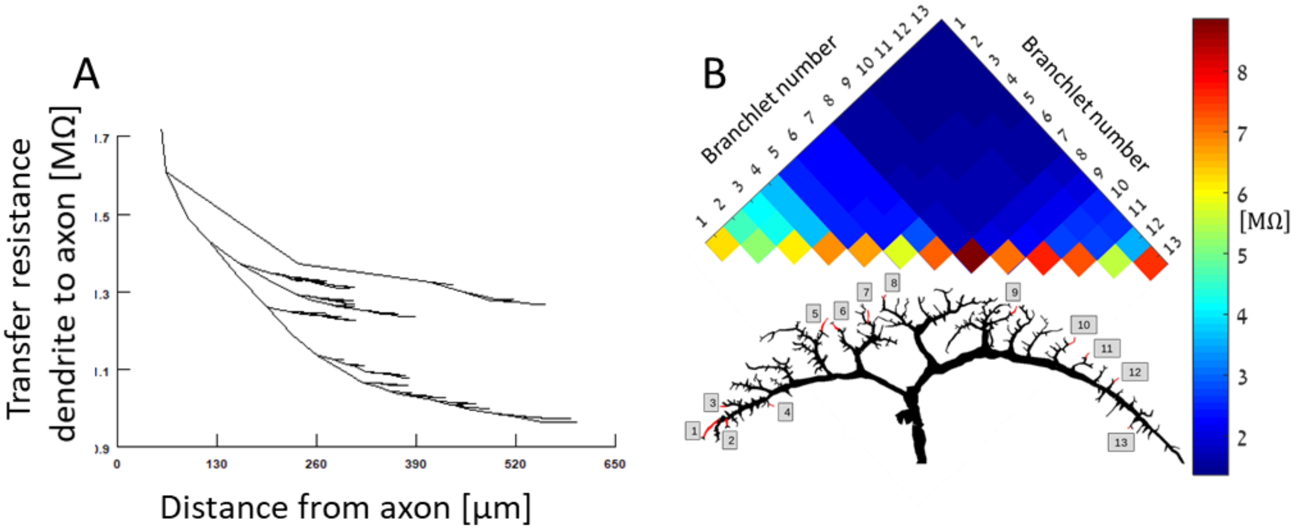

Fig. S1. Approximation of the effect of voltage-dependent membrane ion channels. A. Transfer resistance (V_{axon}/I_{ded}) between each dendritic locus and the axon when the specific membrane resistivity, R_m, is reduced by a factor of 2 (to mimic the opening of voltage-dependent ion channels) as compared to Fig. 3B. The input resistance at the axon (V_{axon}/I_{axon}) is now reduced to 2.2 MΩ. Parameters used were R_m = 1,000 Ω·cm2, R_i = 40 Ω·cm. B. As in Figure 4, but with the reduced R_m value as in A.
